# Restoration of GLP-1 secretion by Berberine is associated with protection of colon enterocytes from mitochondrial overheating in diet-induced obese mice

**DOI:** 10.1038/s41387-018-0061-x

**Published:** 2018-09-24

**Authors:** Yongning Sun, Chenxi Jin, Xiaoying Zhang, Weiping Jia, Jiamei Le, Jianping Ye

**Affiliations:** 1 Department of Traditional Chinese Medicine, Shanghai Jiao Tong University Affiliated Sixth People’s Hospital, Shanghai, 200233 China; 2 Queen Mary School, Nanchang University, Nanchang City, Jiangxi Province 330031 China; 3 Central Laboratory, Shanghai Jiao Tong University Affiliated Sixth People’s Hospital East, Shanghai, 201306 China; 4 Diabetes Institute, Shanghai Jiao Tong University Affiliated Sixth People’s Hospital, Shanghai, 200233 China; 5 Antioxidant and Gene Regulation Laboratory, Pennington Biomedical Research Center, LSU, Baton Rouge, LA 70808 USA

## Abstract

**Objective:**

L-cell dysfunction is reported for GLP-1 reduction in type 2 diabetes. However, the mechanism of dysfunction remains unknown. In this study, we examined mitochondrial function in the mechanistic study in diet-induced obese (DIO) mice.

**Subjects:**

C57BL/6 mice were fed a high-fat diet (HFD) for 16 weeks to establish the DIO model for GLP-1 reduction. The mice were then treated with berberine (BBR) (100 mg/kg/day) for 8 weeks to test the impact on GLP-1 expression. Mitochondrial activities of the colon enterocytes were compared among three groups of mice (lean, DIO, and DIO + BBR) at the end of treatment. Gut microbiota and short-chain fatty acids (SCFAs) were examined to understand the mitochondrial responses. A cellular model treated with palmitic acid (PA) was used in the mechanism study.

**Results:**

A reduction in GLP-1 expression was observed in DIO mice with mitochondrial stress responses in the colon enterocytes. The mitochondria exhibited cristae loss, membrane rupture, and mitochondrial swelling, which was observed with an increase in ATP abundance, complex I activity, and deficiency in the activities of complexes II and IV. Those changes were associated with dysbiosis and a reduction in SCFAs in the colon of DIO mice. In the cellular model, an increase in ATP abundance, loss of mitochondrial potential, and elevation of apoptosis were induced by PA. All of the alterations in DIO mice and the cellular model were attenuated by BBR.

**Conclusion:**

The mitochondrial stress responses were observed in the colon enterocytes of DIO mice for GLP-1 reduction. The stress was prevented by BBR in the restoration of GLP-1 expression, in which BBR may act through direct and indirect mechanisms.

## Introduction

GLP-1 (glucagon-like peptide 1) is a gut hormone, which is a valid target in the control of blood glucose in the treatment of type 2 diabetes. A reduction in plasma GLP-1 contributes to the disorder of blood glucose^1^. Restoration of plasma GLP-1 is a successful strategy in the control of hyperglycemia in the type 2 diabetes patients. Blood GLP-1 is determined by the secretion function of L-cells in the intestine and clearance function in many tissues through DPP-4 (dipeptidyl-peptidase 4)-mediated protein degradation. Inhibition of the clearance process using DPP-4 inhibitors (such as Sitagliptin) is a successful strategy in the induction of plasma GLP-1 in clinics. However, induction of GLP-1 secretion has not been successful in the clinical setting^2^. L-cell dysfunction, not L-cell number reduction, is a mechanism for the decline of plasma GLP-1^3–5^. However, the role of mitochondria in L-cell dysfunction remains unknown. We addressed this issue by studying mitochondria in the colon enterocyte of diet-induced obese (DIO) mice.

Berberine (BBR) is widely used in the treatment of type 2 diabetes in Asian countries^6^. However, the mechanisms remain to be established for the metabolic effects of BBR. Our early studies suggest that BBR downregulates ATP production in mitochondria for activation of AMPK^7^. BBR inhibits the mitochondrial respiration in the inhibition of ATP production, which leads to the elevation of the AMP/ATP ratio^7–9^. BBR was reported to enhance GLP-1 expression in the intestine^10,11^. However, the impact of BBR on mitochondria remains unknown in the GLP-1 regulation. We examined mitochondrial response to BBR in the colon of DIO mice to address this issue.

In this project, we investigated the role of mitochondria in the mechanism of L-cell dysfunction in the colon mucosa. The L-cell dysfunction was found with mitochondrial stress responses in the DIO mice. BBR is able to inhibit the stress responses to preserve the L-cell function.

## Materials and methods

### Chemicals and reagents

Berberine (BBR) was obtained from the National Institute for Food and Drug Control (Beijing, China). The standard chemicals included acetic acid, propionic acid, butyric acid, isobutyric acid, pentanoic acid, isovaleric acid, and 2-methyl valerate. All of the chemicals was more than 98% in purity. Other chemicals included adenosine 5′-diphosphate sodium salt (ADP), fatty acid-free bovine serum albumin (BSA), carbonyl cyanide 4-(trifluoromethoxy) phenylhydrazone (FCCP), oligomycin, antimycin A, rotenone, and succinic acid. Mitochondrial isolation buffer (MSHE + BSA) contains 210 mM mannitol, 70 mM sucrose, 1 mM EGTA, 5 mM HEPES, and 0.5% (w/v) fatty acid-free BSA (pH 7.2). Mitochondrial assay solution (MAS, 1×) contains 220 mM mannitol, 70 mM sucrose, 5 mM MgCl_2_, 10 mM KH_2_PO_4_, 2 mM HEPES, 1 mM EGTA, and 0.2% (w/v) fatty acid-free BSA, pH 7.2 at 37 °C. A 2–3× stock of MAS was prepared for dilution of substrates, ADP, and respiration reagents. Stock solution of succinate (0.5 M) and ADP (1 M) were made in H_2_O and pH 7.2 was adjusted with potassium hydroxide. Stocks of 10 mM FCCP, 2 mM rotenone, 5 mg/ml oligomycin, and 40 mM antimycin A were made in 95% ethanol. All of the chemicals were purchased from Sigma-Aldrich Co. Ltd. (Shanghai, China) unless stated separately. All of the reagents were stored at −20 °C except pyruvate, which was prepared fresh on the day of experiment.

### Animals

The animal experiments were conducted in accordance with the protocol approved by the Institutional Animal Care and Use Committee (IACUC) of Shanghai Jiao Tong University. Male C57BL/6J mice (SPF grade) at 8 weeks in age were obtained from the Shanghai Slac Laboratory Animal Co. Ltd (Shanghai, China). The mice were kept in the animal facility with controlled temperature (20 ± 2 °C), humidity (60 ± 5%), and light cycle at 12 h dark/light. Normal control mice were fed a regular chow diet (NCD, 13.5% calorie in fat; Shanghai Slac Laboratory Animal Co. Ltd). DIO mice (20 mice) were generated by feeding a high-fat diet (HFD, 60% calorie in fat; Research diets, # D12492) for 16 weeks as described elsewhere^12^. DIO mice were divided randomly into two groups: HFD control group (10 mice) and BBR-treated HFD group (10 mice). BBR was delivered through dietary supplementation at 100 mg/kg/day. The mice were treated for 8 weeks with BBR, in which the mice were minored for body weight, food intake, and water intake. Fresh stool samples were collected at 0, 4, and 8 weeks of the treatment and stored at −80 °C immediately for subsequent analysis.

At the end of treatment, mice were subject to tissue collection under anesthesia with intraperitoneal injection of pentobarbital (35 mg/kg). Orbital bleeding was applied in the blood collection. Serum was isolated by centrifugation at 3000 *g* at 4 °C for 10 min and stored at −80 °C until the biochemical assays. The mice were sacrificed by cervical dislocation after blood collection. Livers, colons, and visceral fat tissues were collected and immediately weighed. The tissue samples were rinsed in phosphate-buffered saline (pH 7.4), frozen in liquid nitrogen immediately, and stored at −80 °C until subsequent analysis.

### Histological analysis

The colon tissue samples were fixed in 10% phosphate-buffered formalin acetate at 4 °C overnight and embedded in paraffin wax. Paraffin sections (5 μm) were made and mounted on the glass slides for hematoxylin and eosin (H&E) staining^13^. The paraffin sections of colons were stained by Alcian blue-periodic acid-schiff (AB-PAS) staining according to the manufacturer’s instructions (Solarbio, Beijing, China).

### Glucose tolerance test and blood lipids

Glucose tolerance test (GTT) was performed in the mice after 16 h fasting with peritoneal injection of glucose (2 g/kg). Blood glucose was tested in the tail vein blood at 0, 15, 30, 60, and 120 min using a One Touch glucometer (ACCU-CHEK® performa, Roche). The insulin sensitivity index HOMA-IR [=fasting insulin (mU/l) × fasting glucose (mmol/l)/22.5] was calculated according to the fasting insulin and glucose concentration^7^. Blood lipid profile was examined for serum triglyceride, total cholesterol, high-density lipoprotein cholesterol, and low-density lipoprotein cholesterol using an autoanalyzer (Hitachi 7600-020, automatic analyzer).

### Insulin and GLP-1 assays

Fasting insulin was determined in serum of mice fasted for 6 h with an ELISA kit (Thermo Fisher Scientific, Waltham, MA, USA). GLP-1 was determined in the plasma with the GLP-1 Platinum ELISA kit (BMS2194; eBioscience, California, USA). Blood (300 μl) was collected in tube containing EDTA and DPP-IV inhibitor (1 mM). The plasma was collected after centrifugation at 3000 *g* for 10 min at 4 °C and stored at −80 °C until test. Insulin and GLP-1 data were analyzed with the DENLEY DRAGON Wellscan MK 3 software (Thermo, with Ascent software for Multiskan).

### Microflora assay

Gut microflora was tested using the fecal samples with the 16s ribosomal RNA protocol^14^. The bacterial genomic DNA was prepared and stored at −20 °C prior to the sequence analysis.

Amplification of the bacterial 16s rRNA genes V3–V4 region was performed using the forward primer 338F (5′-ACTCCTACGGGAGGCAGCA-3′) and the reverse primer 806R (5′-GGACTACHVGGGTWTCTAAT-3′) in PCR. PCR products were purified with Agencourt AMPure Beads (Beckman Coulter, Indianapolis, IN) and quantified using the PicoGreen dsDNA Assay Kit (Invitrogen, Carlsbad, CA, USA). After the individual quantification step, amplicons were pooled in equal amounts, and pair-end 2×300 bp sequencing was performed using the Illlumina MiSeq platform with MiSeq Reagent Kit v3 at Shanghai Personal Biotechnology Co., Ltd (Shanghai, China). The Quantitative Insights Into Microbial Ecology (QIIME, v1.8.0) pipeline was employed to process the sequencing data in microbiota analysis. Taxonomical assignments of representative sequences were determined using the RDP classifier with a bootstrap cutoff of 50%.

### Fatty acid assay

The fatty acids were isolated from the frozen fecal samples (0.1 g) and tested using gas chromatography. In assay of short-chain fatty acids (SCFAs), the fecal samples were reconstituted in 0.4 ml distilled water and the supernatant was acidified with a 1/5 volume of 50% H_2_SO_4_ and 1/2 volume of dilution internal standard solution (50 μg/ml), and then extracted with organic solvent ethyl ether. The concentrations of acetic acid, propionic acid, isobutyric acid, butyric acid, valeric acid, and isovaleric acid were determined in the organic phase using gas chromatography (GC-2010; Shimadzu Cooperation, Kyoto, Japan) with a flame ionization detector and an Alltech capillary column (AT-WAX, 30 m × 0.25 mm × 0.25 μm; Alltech Company, ME, USA) operated in the split-less mode. The helium carrier flow was 37.0 cm/s under a column head pressure of 68.0 kPa. The oven temperature was initially 100 °C for 1 min, increased to 170 °C at a rate of 5 °C/min, gradually increased to 230 °C at a rate of 30 °C/min, and maintained for 2 min. The injector and detector temperatures were set at 220 and 250 °C, respectively.

In assay of long-chain fatty acids (LCFAs), the fecal samples were acidified with 1.0 ml 5% H_2_SO_4_ and 100 μg of dilution internal standard solution (19:0 methyl ester, 10 μl from 10 mg/ml), and then was grinded at 60 Hz for 3 min. The homogenate solution was heated at 80 °C  for 90 min. LCFAs were extracted from cooling homogenate with 1.5 ml 0.9% NaCl and 200 μl Hexane. The concentrations of LCFAs were determined in the organic phase using gas chromatography equipped with a flame ionization detector and an DB-5MS column (30 m × 0.25 mm × 0.25 μm; Agilent, USA). The oven temperature was initially 70 °C for 5 min, increased to 200 °C at a rate of 25 °C/min and 240 °C at a rate of 2 °C/min, gradually increased to 300 °C at a rate of 20 °C/min, and maintained for 10 min. The injector and detector temperatures were set at 270 and 320 °C, respectively.

### Quantative real-time PCR (qRT-PCR*)*

Total RNA was extracted from the frozen tissues using the TRIzol RNA isolation reagent (Invitrogen, Carlsbad, CA). Reverse transcription was carried out in 500 ng RNA using the Prime Script^TM^ 1st Strand cDNA Synthesis Kit (TaKaRa, Japan). The qRT-PCR reaction was performed in 20 μl (SYBR Premix Ex TaqTM; TaKaRa Japan) using the applied primer sequences listed in the Supplementary Table 1. The result was normalized against GAPDH mRNA signal.

### Mitochondria and ATP assay

Mitochondria were isolated from the colon tissue by differential centrifugation, and mitochondrial oxygen consumption rates (OCR) were measured in 5 μg using an XF24 Analyzer (Seahorse Bioscience Inc., North Billerica, MA)^15^. For coupling assay, OCR was measured with succinate/rotenone (10 mM/2 μM) in the presence of ADP (4 mM, state 3), oligomycin (2.5 μg/ml, for state 4o), carbonyl cyanide 4-(trifluoromethoxy) phenylhydrazone (FCCP, 4 μM, for state 3u), and antimycin A (4 μM, for nonspecific respiratory background signal). The respiration control rate (RCR) was calculated by state 3/state 4o for the mitochondrial function. The mitochondrial complex assay was performed with 5 μg mitochondria per well, 10 mM pyruvate, 2 mM malate, and 4 mM FCCP. Others  included rotenone (2 mM), succinate (10 mM), antimycin A (4 mM final), and ascorbate plus 1 mM *N*,*N*,*N*9,*N*9-tetramethyl-*p*-phenylenediamine (TMPD, 10 and 100 mM, respectively). ATP level was determined in the fresh tissues with the EnzyLightTM ATP Assay Kit (EATP-100; BioAssay Systems, Northern California, USA).

### Ultrastructure of mitochondria

The fresh colon tissues were rapidly sliced on ice and fixed in 4% glutaraldehyde solution. Specimen of electron microscope was fixed in 1% osmium acid, dehydrated with gradient ethanol, embedded in epoxy resin, and sectioned in ultrathin. The mitochondrial ultrastructure was observed with a transmission electron microscopy (Tecnai G2 Spirit Biotwin/*Tecnai G2 spirit Biotwin, 120kv) .

### Mitochondrial stress responses in cellular model

NCI-H716 cells (CCL-251™; ATCC) were purchased from ATCC, and cultured in 1640 medium with 10% fetal bovine serum without mycoplasma contamination. In the cellular assay, the cells were maintained in Dulbecco's modified Eagleʼs medium supplemented with 0.2% BSA. Mitochondrial stress responses and cell apoptosis were induced by treatment of the cells with palmitic acid (Palmitate) for 24 h at a final concentration of 500 µM. BBR was used at a final concentration of 100 µM to inhibit the cell responses. ATP was determined in the cell lysate using the kit described above. Mitochondrial potential was determined with JC-1 fluorescent dye (Beyotime, Shanghai, China) in combination with the flow cytometry. OCR was determined with the Seahorse technology. Apoptosis was determined with an Annexin V-FITC Apoptosis Detection Kit (Dojindo, Shanghai, China) and cell necrosis was determined with propidium iodide (PI) staining using the flow cytometry.

### Data analysis

The statistical analysis was conducted by excluding the highest and lowest values in the data available in each group to ensure variance similar between groups. The statistical analyses were performed using two-way ANOVA and Student’s *t*-test. Results were expressed as the mean ± SEM. *P-*value < 0.05 was considered statistically significant.

## Results

### Inhibition of obesity and hyperlipidemia by BBR

**Fig. 1:**
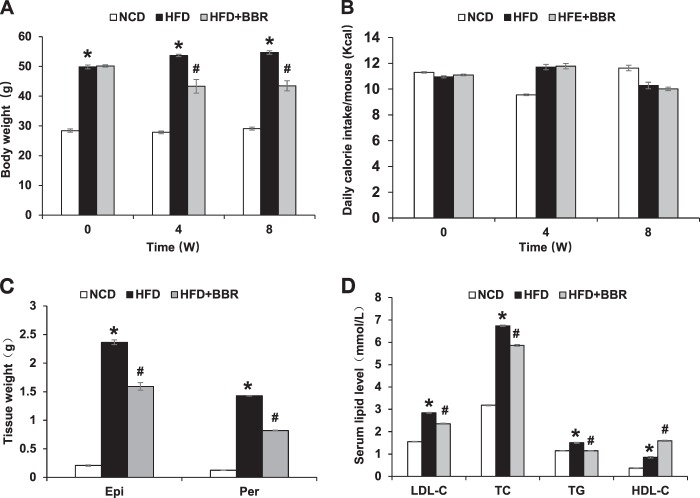
Inhibition of obesity and hyperlipidemia by BBR. **a** Body weight at 0, 4, and 8 weeks of treatment. **b** Average daily calorie intake per mouse. **c** Tissue weight of perirenal fat and epididymal fat pads after 8 weeks of treatment. **d** Serum lipid levels after 8 weeks of treatment. The BBR treatment was administrated for 8 weeks in DIO mice after 16 weeks on high-fat diet. Data are presented as the mean ± SEM (*n* = 6). *P < 0.05 HFD versus NCD, ^#^*P* < 0.05 HFD + BBR versus HFD

DIO mice were generated in C57BL/6 mice with 16 weeks of HFD feeding. BBR was delivered to DIO mice through dietary supplementation for 8 weeks to establish the metabolic impact in the DIO model. The impact included inhibition of weight gain in DIO mice (Fig. 1a), which was observed as early as 4 weeks of treatment without suppression of calorie intake (Fig. 1b). The inhibition was associated with a less gain in fat mass (epididymal and perirenal fat) (Fig. 1c). The impact of BBR was also observed with a reduction in hyperlipidemia with parameters including the low-density lipoprotein C, total cholesterol, and total triglyceride (Fig. 1d). High-density lipoprotein C was increased by the BBR treatment (Fig. 1d). The data suggest that the metabolic activities of BBR were established in the DIO mice.

### Improvement of glucose metabolism by BBR

**Fig. 2:**
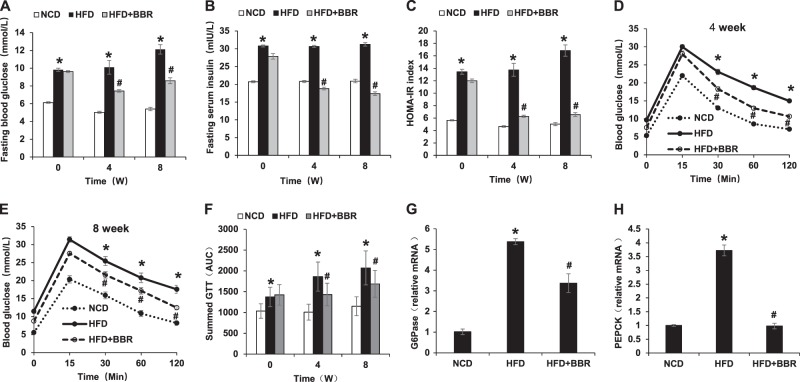
Improvement of insulin sensitivity by BBR. **a** Fasting blood glucose. **b** Fasting serum insulin. **c** HOMA-IR. **d** GTT at 4 weeks of BBR treatment. GTT was performed by intraperitoneal injection of glucose (2 g/kg body weight). **e** GTT at 8 weeks of BBR treatment. **f** Area under the curve of GTT assays. **g** mRNA of G6Pase in liver tissue at 8 weeks of BBR treatment. **h** mRNA of PEPCK in liver tissue at 8 weeks of BBR treatment. Data are presented as the mean ± SEM (*n* = 6). *P < 0.05 HFD versus NCD, ^#^*P* < 0.05 HFD + BBR versus HFD

To test the BBR activity further, glucose metabolism was examined in the mice at the 4 and 8 weeks of the treatment. An improvement in fasting glucose and fasting insulin was observed (Fig. 2a, b), which led to a favorite change of insulin sensitivity index of HOMA-IR (Fig. 2c). The improvement was extended in GTT at 4 and 8 weeks (Fig. 2d–f). Insulin signaling activities was enhanced in the liver as indicated by the reduced expression of the gluconeogenic genes (G6Pase and PEPCK) (Fig. 2g, h). This group of data suggests that the glucose metabolism was improved by BBR in the DIO mice.

### Regulation of L-cells by BBR

**Fig. 3:**
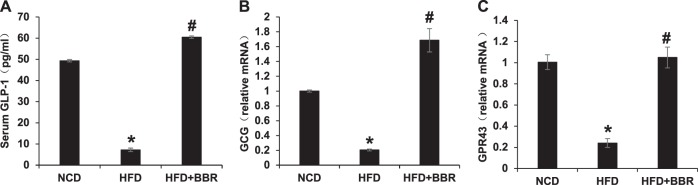
Upregulation of GLP-1 and GPR43 in colon by BBR. **a** Plasma GLP-1. **b** mRNA expression of GCG in colon tissues. **c** mRNA expression of GPR43 in the mouse colon tissues. Data are presented as the mean ± SEM (*n* = 6). *P < 0.05 HFD versus NCD, ^#^*P* < 0.05 HFD + BBR versus HFD

L-cells are located in the epithelial layer of the intestine mucosa and responsible for secretion of the gut hormone GLP-1. A reduction in the serum GLP-1 was observed in DIO mice (Fig. 3a), which was associated with a dramatic decrease in GLP-1 mRNA in the colon tissue (Fig. 3b). These alterations were all corrected in DIO mice treated by BBR (Fig. 3a, b). GPR43 is a receptor of SCFAs in the L-cells, and involved in induction of GLP-1 expression by SCFAs^16^. GPR43 expression was decreased by 80% in DIO mice, and the reduction was also corrected by BBR (Fig. 3c). This group of data suggests that BBR may preserve GLP-1 expression through an impact on L-cells in the colon.

### Mitochondrial damage in colon enterocytes of DIO mice

**Fig. 4:**
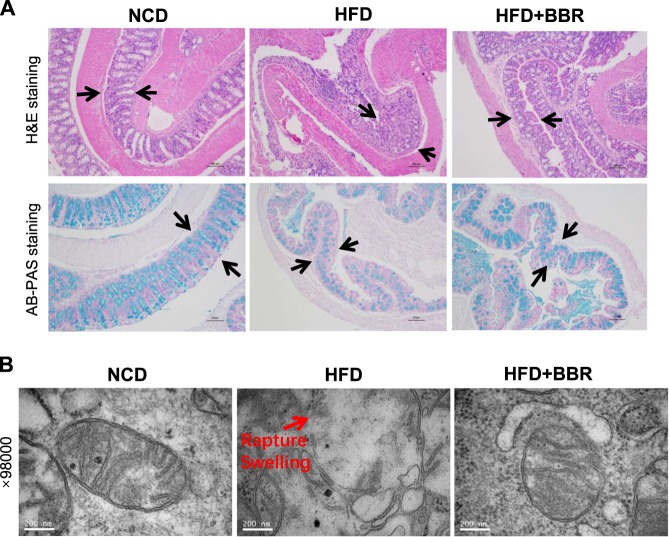
Restoration of mucosal and mitochondrial structure in colon by BBR. **a** Colon tissue histology. Representative fields of H&E staining and AB-PAS staining were taken from the colon tissue slides (×100, scale bars, 100 μm). In HFD mice, the mucosa was damaged and the mucous content was decreased with epithelial cell detached, and these changes were improved by BBR. **b** Mitochondrial structure under the transmission electron microscope. The image was taken at 98,000 times of magnitude

Colon histology and enterocyte mitochondria structure were examined to understand the L-cell dysfunction. The colon tissue integrity and mucus content were examined in the tissue slide under a microscope with H&E staining and AB-PAS staining. Three major histological changes were observed in the DIO mice: loss of mucosa content, loss of epithelial layer in the mucosa surface, and un-even thickness of the mucosal layer (Fig. 4a). Those morphological changes were all improved in DIO mice by BBR.

To understand the mechanism of morphological changes, mitochondria super structure was examined in the enterocytes using the transmission electron microscope. Mitochondria exhibited a structural damage for a loss of cristae and an increase in membrane rupture (Fig. 4b). These changes were associated with a size increase in mitochondria (Fig. 4b), a sign of swelling. The structural changes were corrected in the DIO mice treated by BBR. The structural damage suggests a mechanism for the morphological changes in mitochondria of the colon tissue of DIO mice. Inhibition of the damage by BBR suggests a new activity of BBR in the regulation of enterocyte function.

### Mitochondrial dysfunction in enterocytes

**Fig. 5:**
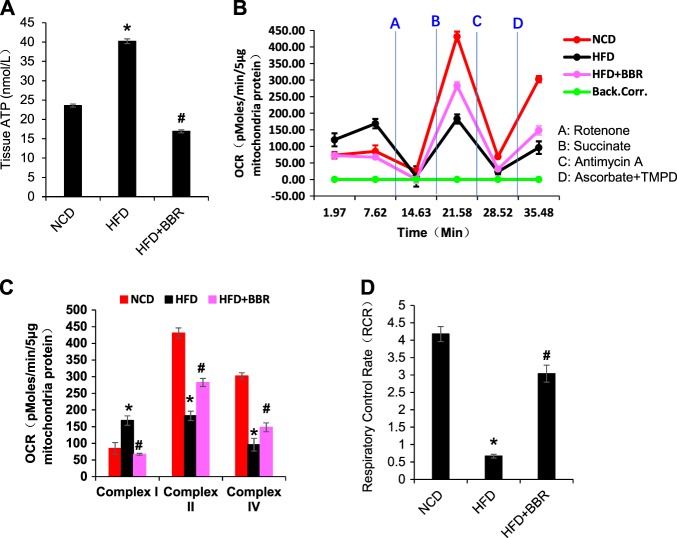
Protection of mitochondrial function in colon by BBR. **a** Total ATP level in the fresh colon tissue. **b** Complex activities of mitochondria. The complex activity was determined by the oxygen consumption rate (OCR) in response to the inhibitors in mitochondria isolated from the fresh colon tissue. The order of inhibitor injection was rotenone, succinate, antimycin A, and ascorbate plus *N*,*N*,*N*9,*N*9-tetramethyl-*p*-phenylenediamine (TMPD). **c** Mean value of maximal OCR in the complex assay. **d** Respiratory control rate (RCR). This was performed with complex II substrate succinate and complex I inhibitor rotenone. Data are presented as the mean ± SEM (*n* = 6). *P < 0.05 HFD versus NCD, ^#^*P* < 0.05 HFD + BBR versus HFD

Mitochondrial function was examined in the colon tissue in the study of L-cell function. Tissue ATP abundance and mitochondrial oxygen consumption were examined in the colon tissue. The ATP abundance was elevated in the homogenization of fresh colon tissue of the DIO mice (Fig. 5a), suggesting that ATP production exceeded ATP demand in the enterocytes of DIO mice. In the isolated mitochondria, the complex activities were tested by measuring mitochondrial OCR using the Seahorse technology. The complex I activity was increased, but the complex II and IV activities were decreased in the DIO mice (Fig. 5b, c). When the oxygen consumption was tested under the complex II substrate, the RCR of mitochondria was dramatically decreased in DIO mice, and partially restored by BBR (Fig. 5d). The combination of ATP elevation with complex I super-activation suggests that mitochondria were under a stress response in the DIO mice. The stress responses were corrected by BBR (Fig. 5a–d). These data suggest that mitochondrial stress may lead to the structural damage in DIO mice, which were all attenuated by BBR.

### Induction of mitochondrial stress by fatty acids

**Fig. 6:**
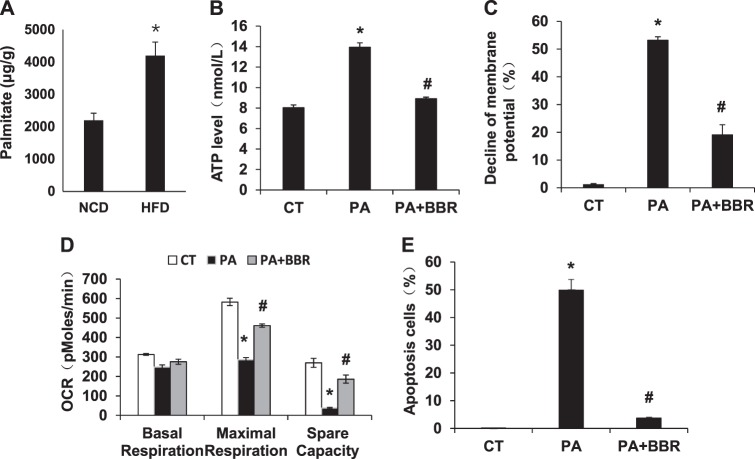
Regulation of mitochondrial stress and apoptosis in palmitate-treated cells by BBR. Palmitate was used to represent the long-chain fatty acid palmitic acid in the induction of mitochondrial stress responses in the intestine epithelial cell line NCI-H716. **a** Palmitate level in the large intestine. Palmitate was determined in the fecal samples. **b** ATP elevation in palmitate-treated cells. **c** Decline of mitochondrial potential in palmitate-treated cells. **d** Reduction in mitochondrial function in palmitate-treated cells. **e** Apoptosis in response to palmitate. Data are presented as the mean ± SEM (*n* = 3). *P < 0.05 PA versus control; ^#^*P* < 0.05 PA + BBR versus PA

To understand the cause of mitochondrial stress, we explored the effect of LCFAs on cell responses in a cellular model, in which the intestine epithelial cell line NCI-H716 was treated with palmitate (for palmitic acid). The study design was based on the elevation of LCFAs and reduction of GLP-1 secretion in the colon of DIO mice^17^. Palmitate level was increased in the large intestine by HFD (Fig. 6a). In response to palmitate treatment, ATP abundance was increased, and mitochondrial potential was decreased in the cellular model (Fig. 6b, c). Mitochondrial maximal respiration was decreased and cell apoptosis was elevated in the same condition (Fig. 6d, e). Cell necrosis was not observed in the model with PI staining of the dead cells (data not shown). When BBR was added into the cell culture, all of the changes were dramatically reduced or blocked (Fig. 6b, e). The data suggest that the mitochondrial stress was induced by palmitate and inhibited by BBR.

### Regulation of SCFAs and gut microflora by BBR

**Fig. 7:**
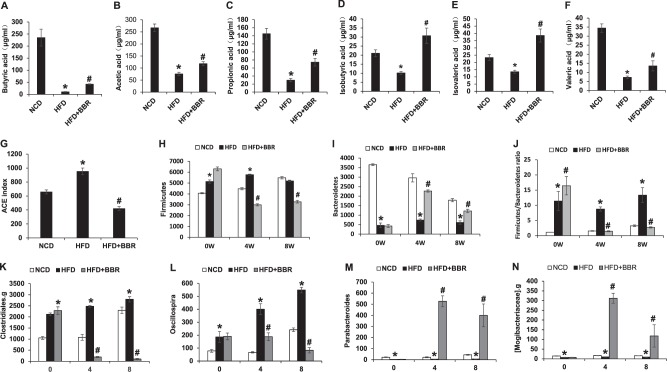
Regulation of SCFAs and gut microflora by BBR. **a**–**f** The concentration of butyric acid, acetic acid, propionic acid, isobutyric acid, isovaleric acid, and valeric acid in feces of HFD mice after 8 weeks of BBR treatment. **g** Bacteria diversity in feces as indicated by ACE richness. **h**, **i** The abundance of Firmicutes and Bacteroidetes. **j** The Firmicutes/Bacteroidetes ratio. **k–n** The abundances of Clostridiales.g, Oscillospira, Parabacteroides, and Mogibacteriaceae.g. Data are presented as the mean ± SEM (*n* = 6). *P < 0.05 HFD versus NCD, ^#^*P* < 0.05 HFD + BBR versus HFD

SCFAs are produced in the large intestine by microflora-mediated fermentation of dietary fibers, and used by enterocytes as an energy source. Among SCFAs, butyrate (sodium salt of butyric acid) was reported to improve glucose metabolism^18^ and induce GLP-1 expression in L-cells^19^. A reduction in SCFAs may contribute to the mitochondrial stress in DIO mice. To test the possibility, six types of SCFAs (butyric acid, acetic acid, propionic acid, isobutyric acid, isovaleric acid, and valeric acid) were examined in the feces of mice. In the control lean mice, butyric, acetic and propionic acids were most abundant among the six SCFAs in the feces with concentrations between 140 and 260 µg/ml in the normal control mice (Fig. 7a–c), which were 3–5-fold higher than those of other three SCFAs of 20–35 µg/ml (Fig. 7d–f). In DIO mice, all of the six SCFAs were significantly reduced relative to the control mice (Fig. 7a–f). The most reduction was observed in butyric acid. The reduction was partially blocked by BBR in DIO mice, but remained significantly low especially in the three dominant SCFAs (butyric acid, acetic acid, and propionic acid). The data suggest that BBR may not have a significant effect on the regulation of SCFAs in DIO mice in this study. In the same condition, a dramatic improvement in L-cell function was observed with a little recovery in SCFAs, suggesting that SCFAs may not be an essential factor in the BBR activity.

The gut microbiota was examined in the fecal samples to understand the basis of the SCFA alterations. The analysis was conducted using the 16s ribosomal RNA protocol^14^. An increase in microbiota diversity (richness and evenness) was observed with the Ace estimator in DIO mice (Fig. 7g). At the phylum level, *Firmicutes* and *Bacteroidetes* are the most abundant bacteria in the gut microflora. Firmicutes was increased, and Bacteroidetes was decreased in DIO mice (Fig. 7h, i). The ratio of Firmicutes to Bacteroidetes, a widely used marker of gut dysbiosis^20^, was increased by 10-folds in DIO mice (Fig. 7j). At the genus level, the relative abundance of Clostridiales.g and Oscillospira were increased by HFD (Fig. 7k, l). All of the alterations were improved in DIO mice by BBR (Fig. 7a–l). The relative abundance of Parabacteroides and Mogibacteriaceae.g were decreased in DIO mice and the decrease was reversed by BBR well above the normal level (Fig. 7m, n). The data suggest that BBR is able to correct dysbiosis in DIO mice.

## Discussion

Protection of L-cell function represents a strategy in the promotion of GLP-1 secretion in favor of treatment of type 2 diabetes^2,21,22^. L-cell dysfunction has been reported in the DIO models for the GLP-1 reduction^3–5^. Although LCFAs have been reported to inhibit L-cell function through induction of endoplasmic reticulum stress or cell apoptosis^23,24^, the mitochondrial response in the L-cell dysfunction remains unknown. Our data suggest that the L-cell dysfunction is associated with mitochondrial stress responses. The stress was observed with alterations in structure and function of mitochondria. The structural alteration included cristae loss, membrane rupture, and mitochondrial swelling, which were observed together with an increase in ATP and a decrease in the respiration function. The stress was observed in the cellular model in response to the challenge by saturated LCFA (palmitic acid), suggesting a mechanism of L-cell dysfunction in the colon. The stress was inhibited by BBR in vitro and in vivo. Those data suggest that L-cell dysfunction may be a result of mitochondrial stress upon the challenge by LCFAs in the DIO model. The stress was associated with cell apoptosis (but not necrosis), which was consistent with the L-cell apoptosis reported in the DIO model and in GLP-1 secretion cell line^3,23^.

Our data suggest that the mitochondrial stress is characterized by ATP overproduction. Palmitic acid is known to induce cell apoptosis through induction of reactive oxygen species (ROS)^25^. However, the mechanism of ROS production was not identified in the study. Current study suggests that ATP elevation triggers the ROS production. The ATP elevation was observed in the colon tissues of DIO mice. The elevation is likely a result of over-supply of the LCFAs as suggested by the data of cellular model. The overproduction is supported by the activation of complex I in the colon tissue, which is a common mechanism in the control of ATP overproduction upon fuel over-loading in mitochondria^26,27^. The complex I is the major component in the production of ROS in mitochondria^28^. ROS is able to reduce ATP production through a decrease in the mitochondrial potential^26,27^. These data suggest that the complex I activation is a result of ATP overproduction in the colon. In addition, the ATP elevation may involve in a reduction in ATP hydrolysis following mitochondrial damage by ROS in some of the enterocytes. Mitochondria hydrolyze ATP to prevent a decrease in membrane potential in the physiological conditions^29^. When the ATP hydrolysis was inhibited by mitochondrial damage, ATP consumption is decreased to promote the ATP elevation. In vivo, infiltration of immune cells into the intestine may also contribute to the ATP elevation of DIO mice^3^.

Our data suggest that BBR may control the mitochondria stress by prevention of ATP overproduction, which is called “mitochondrial overheating” in the current study. Mitochondria produce superoxide in response to ATP overproduction, in which superoxide is able to open the mitochondrial permeability transition pore to reduce the mitochondrial potential to control ATP production^27^. In such a stress condition, ROS may abolish the mitochondrial potential through a damaging effect on the mitochondrial membrane. ATP overproduction is considered as a trigger of mitochondrial stress responses in the current study. The overproduction was downregulated by BBR in vitro and in vivo, which was coupled with prevention of the mitochondrial damage. The data suggest that BBR protected the mitochondria by suppression of ATP overproduction in DIO mice. The observation is consistent with the BBR activity in the suppression of oxidative phosphorylation in mitochondria^7,8^. These data suggest that BBR may protect L-cells by prevention of the mitochondrial overheating through a direct effect on mitochondria.

SCFAs may contribute to the BBR activity in the regulation of GLP-1 in vivo. BBR was reported to regulate the lipid metabolism through an impact on SCFAs^30^. In L-cells, SCFAs induce GLP-1 expression and secretion by stimulation of GPR43, which are supported by the effects of dietary fibers^31,32^. GPR43 gene knockout (FFAR2^−/−^) mice exhibited a reduction in GLP-1 secretion in vivo and in vivo^16^. In addition, SCFAs induce L-cell differentiation from progenitors^21,33^. In the current study, SCFAs were modestly up-regulated in DIO mice by BBR probably through an impact on dysbiosis. These studies suggested that SCFAs may contribute to the BBR effect on GLP-1 secretion. BBR was effective in the regulation of mitochondria in the cellular model in the absence SCFAs, which suggests that BBR may regulate GLP-1 expression independently.

In summary, the study demonstrates that mitochondria of colon enterocytes suffered a series of stress responses in the DIO mice for reduction in GLP-1 secretion. Oral administration of BBR inhibited the mitochondrial stress responses to restore the GLP-1 secretion in the mice. The mechanisms likely involve direct and indirect effect of BBR. The direct effect is down-regulation of the mitochondrial ATP production under the condition of LCFA challenge. The indirect includes correction of SCFAs and dysbiosis, which may contribute to the BBR activity in vivo.

## Electronic supplementary material

Supplementary Table 1
