# Supplementary material for: Restoration of GLP-1 secretion by Berberine is associated with protection of colon enterocytes from mitochondrial overheating in diet-induced obese mice
Source: Nutr Diabetes. 2018 Sep 24;8:53. doi: 10.1038/s41387-018-0061-x (PMC6155143; doi:10.1038/s41387-018-0061-x)
Supplement: Supplementary file 1 — Supplementary Table 1 [file 41387_2018_61_MOESM1_ESM.docx]

**Supplement 1**

Suppl. 1 qRT-PCR primer sequence

| Primer name | Primer sequence (5'to3') | |
| --- | --- | --- |
|  | Forward primer | Reverse primer |
| GPR43 | CACGCAGGGGAGAGGATCTG | GTCTGGGGTCATTCTCCTTGG |
| Gcg | ACGCCCTTCAAGACACAGAG | GGCAATGTTGTTCCGGTTCC |
| PEPCK | AGTTCGTGGAAGGCAATGCT | CTTCAGCTTGCGGATGACAC |
| G6Pase | GCAGTGGTCGGAGACTGGTT | ATAGGCACGGAGCTGTTGCT |
| GAPDH | AGGTCGGTGTGAACGGATTTG | TGTAGACCATGTAGTTGAGGTCA |

**Supplement 2**

Suppl. 2.1 Descriptive Statistics of HFD and HFD+SA groups

|  | Group | Time(W) | Mean | Std. Deviation | N |
| --- | --- | --- | --- | --- | --- |
| weight (g) | HFD | 0 | 48.500 | 2.3333 | 10 |
|  |  | 4 | 52.550 | 1.7070 | 10 |
|  |  | 8 | 53.250 | 2.1246 | 10 |
|  |  | Total | 51.433 | 2.9206 | 30 |
|  | HFD+SA | 0 | 47.850 | 2.3576 | 10 |
|  |  | 4 | 48.550 | 1.9358 | 10 |
|  |  | 8 | 48.500 | 2.2236 | 10 |
|  |  | Total | 48.300 | 2.1278 | 30 |
|  | Total | 0 | 48.175 | 2.3072 | 20 |
|  |  | 4 | 50.550 | 2.7140 | 20 |
|  |  | 8 | 50.875 | 3.2277 | 20 |
|  |  | Total | 49.867 | 2.9857 | 60 |
| HOMA-IR | HFD | 0 | 11.999570400 | 1.7216388306 | 10 |
|  |  | 4 | 11.953822900 | 2.9933280735 | 10 |
|  |  | 8 | 14.281906850 | 2.3757027924 | 10 |
|  |  | Total | 12.745100050 | 2.5834302515 | 30 |
|  | HFD+SA | 0 | 12.647846200 | .9579017148 | 10 |
|  |  | 4 | 9.081475490 | 1.0377419621 | 10 |
|  |  | 8 | 10.380759650 | 1.4203267522 | 10 |
|  |  | Total | 10.703360447 | 1.8686375153 | 30 |
|  | Total | 0 | 12.323708300 | 1.3961573427 | 20 |
|  |  | 4 | 10.517649195 | 2.6316313755 | 20 |
|  |  | 8 | 12.331333250 | 2.7629729788 | 20 |
|  |  | Total | 11.724230248 | 2.4610254577 | 60 |

Suppl. 2.2 The two way ANOVA analysis of HFD and HFD+SA groups

| Source | | Dependent Variable | Type III Sum of Squares | df | Mean Square | F | Sig. |
| --- | --- | --- | --- | --- | --- | --- | --- |
| Corrected Model | | weight (g) | 281.833a | 5 | 56.367 | 12.469 | .000 |
|  |  | HOMA-IR | 163.124b | 5 | 32.625 | 9.071 | .000 |
| Intercept | | weight (g) | 149201.067 | 1 | 149201.067 | 33006.381 | .000 |
|  |  | HOMA-IR | 8247.454 | 1 | 8247.454 | 2293.101 | .000 |
| Group | | weight (g) | 147.267 | 1 | 147.267 | 32.578 | .000 |
|  |  | HOMA-IR | 62.531 | 1 | 62.531 | 17.386 | .000 |
| Time(W) | | weight (g) | 86.908 | 2 | 43.454 | 9.613 | .000 |
|  |  | HOMA-IR | 43.676 | 2 | 21.838 | 6.072 | .004 |
| Group * Time(W) | | weight (g) | 47.658 | 2 | 23.829 | 5.272 | .008 |
|  |  | HOMA-IR | 56.917 | 2 | 28.459 | 7.913 | .001 |
| Error | | weight (g) | 244.100 | 54 | 4.520 |  |  |
|  |  | HOMA-IR | 194.218 | 54 | 3.597 |  |  |
| Total | | weight (g) | 149727.000 | 60 |  |  |  |
|  |  | HOMA-IR | 8604.797 | 60 |  |  |  |
| Corrected Total | | weight (g) | 525.933 | 59 |  |  |  |
|  |  | HOMA-IR | 357.342 | 59 |  |  |  |
|  |  |  |  |  |  |  |  |

The result of two way ANOVA analysis suggest that the administration of SA induced a significant reduction (P<0.05) in body weight and HOMA-IR in DIO mice (N=10).

**Supplement 3**

A B


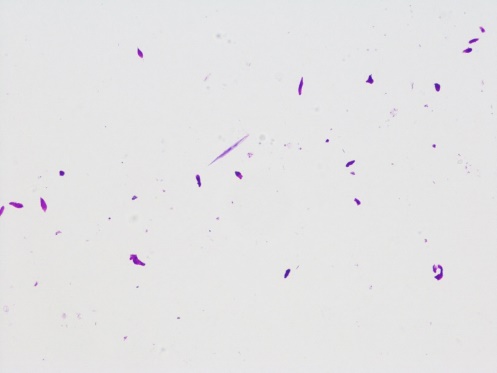

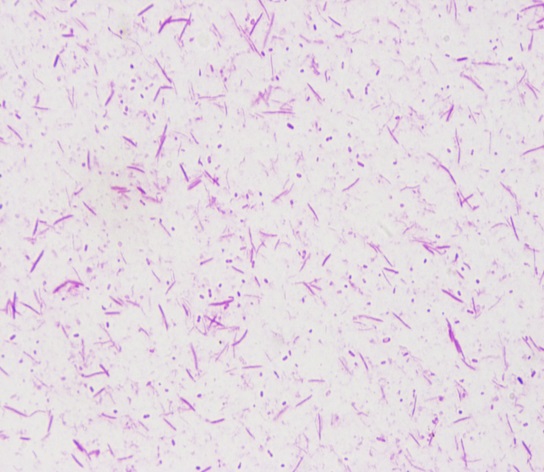

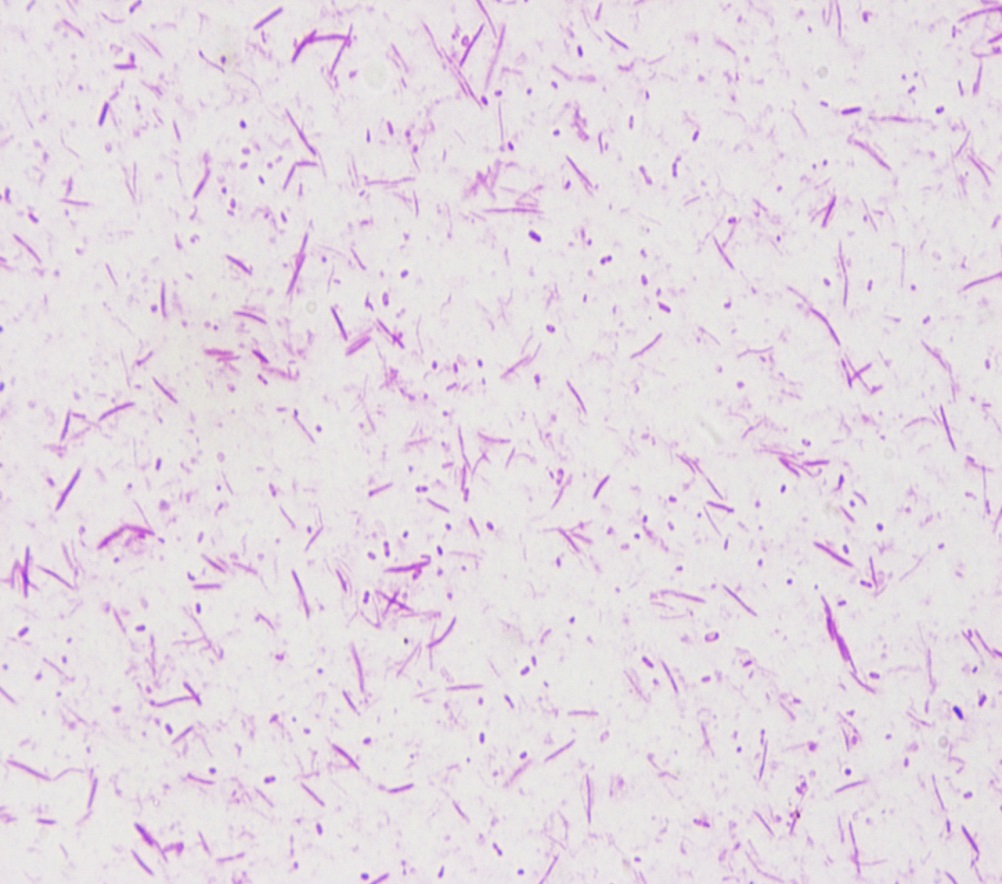

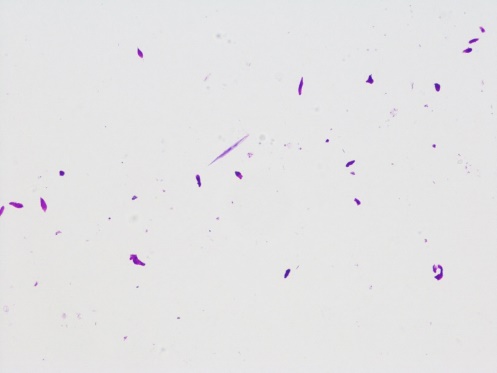


Suppl. 3 Gram stained bacteria in mouse feces. (A) Fecal sample of HFD mice. Many Gram positive bacteria are found in the feces of control DIO mice (pointed by arrows). The bacteria were in purple color. (B) Fecal sample of pseudo-germ-free HFD mice (P-HFD). No Gram positive bacteria are found, but Gram-positive bacteria-like particles are observed with a difference in particle size and shape (arrows). PGF status was confirmed in the mice model.

**Supplement 4**

*

**#**

Suppl. 1 The ratio of TG/HDL-C. Data are presented as the mean ± SEM (n = 10). * *p* < 0.05 HFD versus NCD, #P < 0.05 HFD+SA versus HFD. The ratio was decreased by HFD and was not improved by SA treatment. HDL-C was reported to be increased (Jiefei Bai, et al. Int J Clin Exp Pathol, 8:13193-13200, 2015), decreased (Sun, et al. Lipids in Health and Disease, 16:145, 2017) and unchanged (Yuling Ding, et al. EXCLI Journal, 16:328-339, 2017) in literature. Our results suggest that HDL-C was increased in DIO mice. This issue has been discussed in the discussion section.

**Supplement 5**

Suppl. 5 The TG levels of feces. TG (mMol/g) was determined in the fecal samples and normalized with fecal wet weight (gram). Data are presented as the mean ± SEM (n = 3). * *p* < 0.05 HFD versus NCD, ^#^ *p* < 0.05 HFD+SA versus HFD.
